# Novel lncRNA LncMSTRG.11341.25 Promotes Osteogenic Differentiation of Human Bone Marrow Stem Cells via the miR-939-5p/PAX8 Axis

**DOI:** 10.34133/research.0601

**Published:** 2025-02-06

**Authors:** Feifei Ni, Jianjun Li, Qin Yin, Yangyang Chen, Zengwu Shao, Hong Wang

**Affiliations:** ^1^ Department of Orthopedics, Union Hospital, Tongji Medical College, Huazhong University of Science and Technology, Wuhan 430022, China.; ^2^ Department of Orthopaedics, Shengjing Hospital of China Medical University, Shenyang 110004, Liaoning, China.; ^3^ Department of Orthopedics, Wuxi Ninth People’s Hospital Affiliated to Soochow University, Wuxi 214000, Jiangsu, China.; ^4^ Department of Orthopedic, The First Affiliated Hospital of Zhengzhou University, Zhengzhou 450052, China.

## Abstract

Human bone marrow stem cells (hBMSCs) play an important role during the fracture healing phase. Previous clinical studies by our research group found that fracture healing time was obviously delayed in patients who underwent splenectomy, for combined traumatic fractures and splenic rupture, which is most likely related to the dysregulation of immune inflammatory function of the body after splenectomy. A large number of studies have reported that the inflammatory factor interleukin-1β plays an important role in the multi-directional differentiation ability and immune regulation of BMSC, but its specific regulatory mechanism needs to be further studied. Recently, long noncoding RNAs (lncRNAs) have attracted remarkable attention owing to their close relationship with stem cell osteogenesis and potential role in various bone diseases. In this study, we explored the molecular mechanism of a novel lncRNA, LncMSTRG.11341.25 (LncMSTRG25), in terms of its effects on osteogenic differentiation of hBMSCs. Our results reveal significant up-regulation of LncMSTRG25, osteogenic differentiation markers during the osteogenic differentiation of hBMSCs, and decreased expression of miR-939-5p with an increase in differentiation time. LncMSTRG25 knockdown significantly inhibited the osteogenic ability of hBMSCs. When we knocked down PAX8 alone, we found that the osteogenic ability of hBMSCs was also significantly reduced. The interaction between LncMSTRG25 and PAX8 was verified using the RNA immunoprecipitation assay, RNA pull-down assays, silver staining, and the dual-luciferase reporter. The results show that LncMSTRG25 can function as a sponge to adsorb miR-939-5p, inducing the osteogenic differentiation of hBMSCs by activating PAX8. These findings deepen our understanding of the regulatory role of lncRNA–miRNA–mRNA networks in the immune microenvironment of bone marrow, and highlight the important role played by the spleen as an immune organ in fracture healing.

## Introduction

Splenic rupture combined with limb fracture is a common multiple abdominal injury in clinical practice, and total splenectomy is the standard treatment. The spleen is the largest immune organ in the body; it is rich in immune cells and is the site of immune cell-mediated immune responses such as macrophages, B lymphoid cells, and T lymphoid cells, which are crucial to the immune function of the body. Splenectomy leads to an imbalance in immune regulation and a decrease in immune surveillance function [1]. There are few reports on the effect of splenectomy on fractures. Previous research efforts have focused on the role of osteogenic factors in the fracture healing process, and current research focuses on the mechanisms of immune function changes in different stages of fracture healing [2,3]. Our previous clinical study found that fracture healing is significantly delayed in patients with a fracture combined with splenic rupture who underwent splenectomy; moreover, inflammatory-related factors such as interleukin-6 (IL-6), tumor necrosis factor-α (TNF-α), and IL-1β are significantly reduced in the acute fracture stage [4–6]. These studies have shown that the inflammatory response at the fracture site is indispensable during certain stages of bone healing [7]. There is increasing evidence that IL-1β can affect the function of marrow stem cells (MSCs) [8], including multi-directional differentiation capacity and immune adjustment, but the specific regulatory mechanisms require further study.

Stem cells are multipotent cells that originate from the early developing mesoderm, and include umbilical cord MSC cells, adipose MSCs, dental pulp, and bone marrow stem cells. These cells have regenerative capacity and can be induced to differentiate into osteoblasts, chondrocytes, and adipocytes [9,10]. At the same time, MSCs have strong properties of tissue regeneration and organ repair, which play important roles in fracture healing, osteoporosis, and infection. Moreover, bone marrow stem cell (BMSC) differentiation plays an important role in fracture healing development [11].

Long noncoding RNAs (lncRNA) are a group of mRNA, having a length of >200 nucleotides, poly(A) tail, and promoter structure, and which are dynamically expressed during differentiation [12]. LncRNAs have been shown to regulate various cellular differentiations, participate in MSC differentiation, and contribute significantly to osteogenesis. LncRNA may pass through the microRNA (miRNA) “sponge” effect and impact downstream gene expression; the mechanism of action has become a hot topic for new clinical practical targets [13,14]. miRNA is critical for tissue development, binding either fully or partially complementarily to the 3′ untranslated region (3′UTR) of target gene mRNA [15]. Studies have reported that miRNAs, including miR-1260a, miR-210, and miR-19a/b [16–18], play important roles in regulating osteogenesis. At present, with a gradual understanding of lncRNA, the relationship between lncRNA, miRNA, mRNA, and oogenesis remains to be elucidated.

**Fig. 1.**
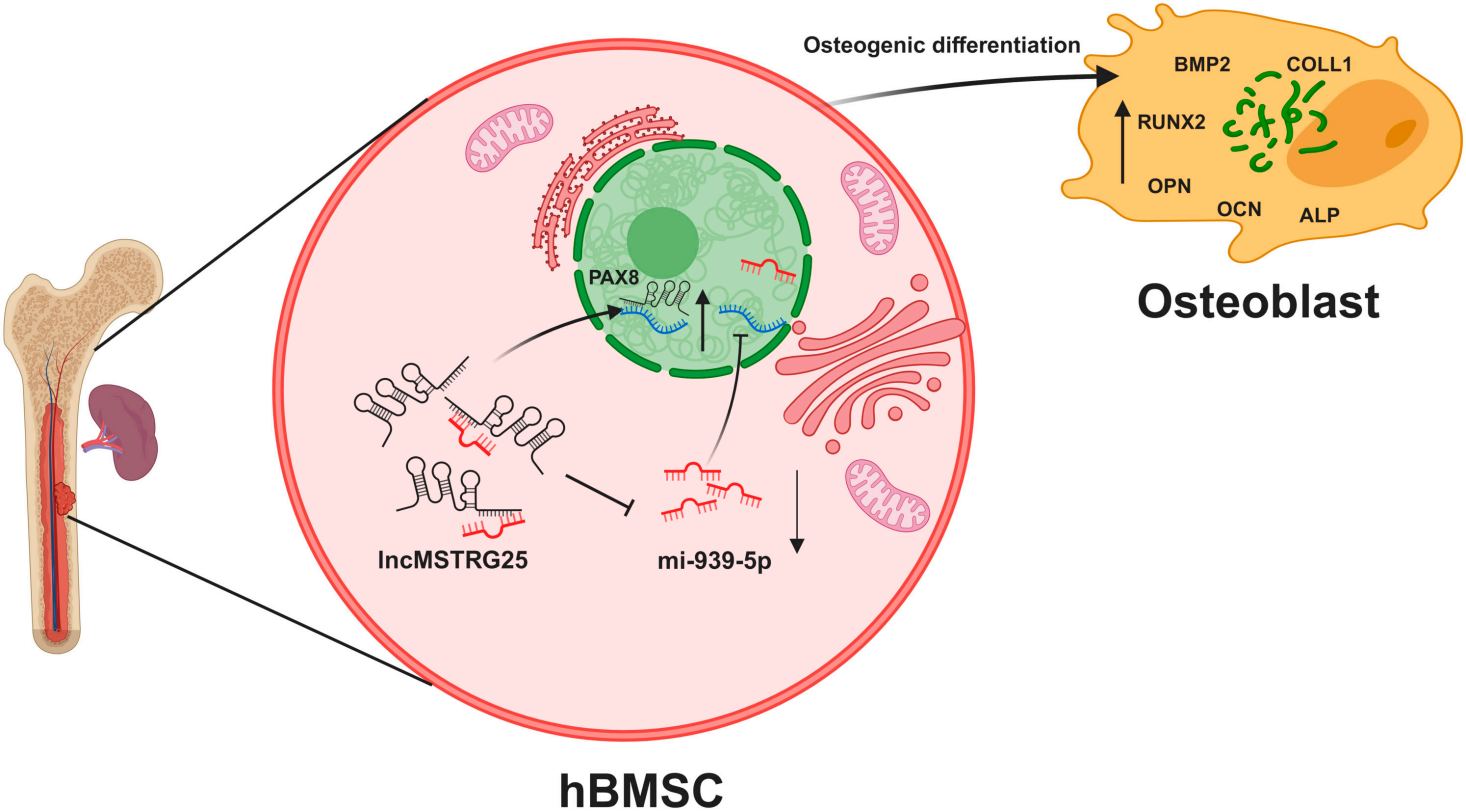
Model of LncMSTRG25 regulation of hBMSC osteogenic differentiation.

Research indicates that IL-1β facilitates the healing of traumatic fractures [19,20]. Therefore, we hypothesized that gene expression changes induced by IL-1β after fracture combined with splenectomy may serve as a key factor in the osteogenic differentiation of human bone marrow stem cells (hBMSCs). Therefore, we investigated whether the transcriptional profile of IL-1β facilitates the differentiation of hBMSCs into osteoblasts. Through high-throughput RNA sequencing at the cellular level, we found that an lncRNA [LncMSTRG.11341.25 (LncMSTRG25)] and PAX8, whose mRNA is coexpressed with it, showed an increase during osteogenic differentiation. We first explored LncMSTRG25-positive functions in regulating hBMSC osteogenic differentiation and its potential mechanism. We conducted relevant experiments to analyze the interaction between LncMSTRG25 and PAX8 and the effects on hBMSCs. In addition, LncMSTRG25, miR-939-5p, and miR-939-5p, in turn, regulate the expression of the target gene PAX8, further regulating hBMSC osteogenic differentiation. Our data suggest that LncMSTRG25 promotes bone regeneration and fracture healing, which further confirms that the immune microenvironment of the spleen is vital to the fracture healing process. The results of this study provide a new theoretical basis for spleen preservation after fracture combined with spleen rupture (Fig. 1).

## Results

### Differentially expressed genes in hBMSC osteogenetic differentiation

**Fig. 2.**
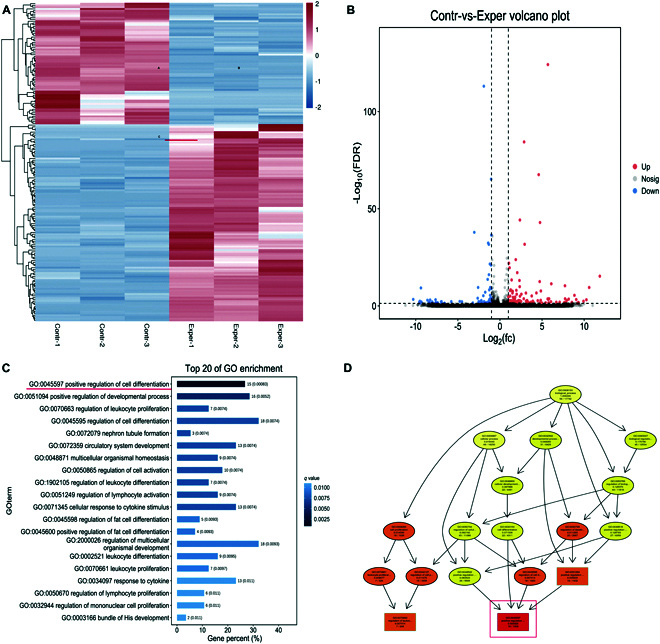
Differentially expressed genes in hBMSC osteogenetic differentiation. (A) Cluster heat map of differentially expressed lncRNAs between the osteogenic differentiation IL-1β group and control group. *P* < 0.05 and fold change > 2 applied as criteria for screening genes with marked changes in expression. (B) Statistics of differentially expressed genes; 112 genes were up-regulated and 69 were down-regulated. (C) lncRNA–mRNA GO enrichment analysis showed that the up-regulated genes might be associated with different biological processes, among which the biological process with the highest enrichment is shown. (D) Each GO term is enriched, and the 10 most significant nodes are represented by rectangles, the colors of which represent the significance of enrichment (increasing from yellow to red).

We determined the characteristics of the lncRNA transcript by comparing hBMSCs with IL-1β and control groups. To detect differentially expressed transcripts between samples and groups, the edgeR package was employed (www.bioconductor.org/packages/release/bioc/html/edgeR). HISAT2 [21] adoption of global and local search methods can be more effective than RNA sequencing data of spliced reads and is now the most accurate software with the highest ratios. Based on the results of differential analysis, using false discovery rate (FDR) < 0.05 and |log2FC| > 1, a total of 181 lncRNAs were significantly differentially expressed in hBMSCs induced by IL-1β osteogenesis; of these, 112 lncRNAs were up-regulated and 69 were down-regulated (Fig. 2A and B). Gene Ontology (GO) differential gene enrichment analysis showed that IL-1β osteogenic induction primarily focused on the biological process of positively regulating cell differentiation and GO term 0045597 (Fig. 2C and D).

### LncMSTRG25, miR-939-5p, and PAX8 expression in hBMSCs

**Fig. 3.**
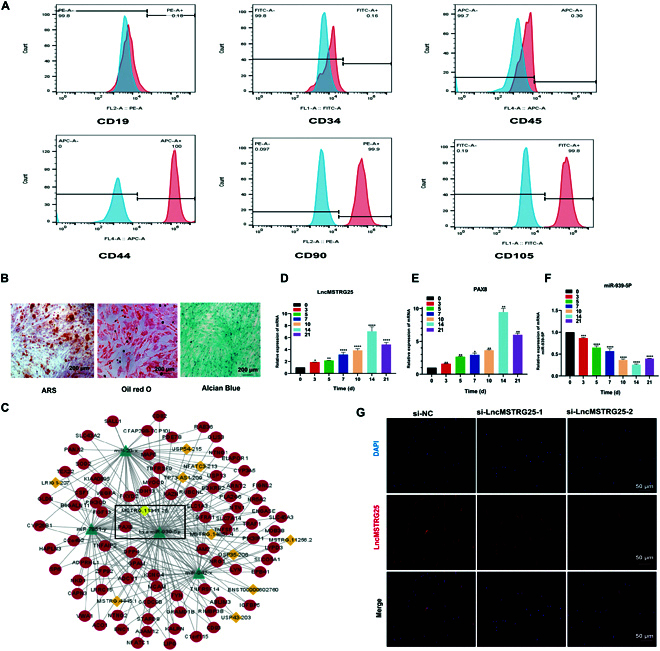
hBMSC identification and gene expression in hBMSCs. (A) Flow cytometry analysis of hBMSC surface markers. Antibodies against CD19, CD34, CD44, CD45, CD73, and CD90 were used for flow cytometry. (B) ARS, Oil Red O staining, and Alcian Blue staining results showing the osteogenic, adipogenic, and chondrogenic differentiation abilities of hBMSCs. (C) Bioinformatics prediction of LncMSTRG25 and the predicted mRNA and miRNA targets. (D to F) PCR detection of the relative expression of LncMSTRG25, PAX8, and miR-939-5p on days 0, 3, 5, 7, 10, 14, and 21. (G) FISH results showed that LncMSTRG25 was localized to the cytoplasm. Data are the mean ± SD of 3 independent experiments. *P < 0.05, **P < 0.01, ***P < 0.001, and ****P < 0.0001.

Through flow cytometry, hBMSCs were confirmed to express low levels of CD19, CD34, and CD45 while showing high expression of CD44, CD90, and CD105 (Fig. 3A). Next, we cultured hBMSCs in osteogenic medium to induce osteogenic differentiation and evaluated their potential using Alizarin red S (ARS) (Fig. 3B). For hBMSCs in terms of multi-directional differentiation ability, we observed that the accumulation of lipids increased, lipid droplets gradually increased, and the fat differentiation potential was promoted (Fig. 3B). Alcian blue staining showed that the hBMSCs had the potential to differentiate into cartilage cells (Fig. 3B). We used the RNAplex [22] base dior to identify short interactions between 2 long-chain RNA software packages in order to predict and complement the mRNA antisense lncRNA combination, which contains the ViennaRNA package [23]. Three software packages—miReap, miRanda, and TargetScan—were used to predict miRNA–lncRNA interactions, and miRNA sequences and family data were retrieved from the TargetScan website (www.targetscan.org). The lncRNA–miRNA–mRNA network was built by compiling all coexpressions and competing triplets, and was visualized using Cytoscape V3.6.0 (http://www.cytoscape.org/). Five lncRNAs satisfied the requirements of the above 3 simultaneously. The purpose of this study was to identify the expression of targets by inhibiting and expressing these targets to observe their effects on osteogenesis. Therefore, a novel lncRNA, LncMSTRG25, and its coexpression with mRNA PAX8 and miRNA miR-939-5p attracted our attention (Fig. 3C). We first verified the up-regulation of LncMSTRG25 and PAX8 and the sustained down-regulation of miR-939-5p during osteogenesis using quantitative polymerase chain reaction (qPCR) (Fig. 3D to F). LncMSTRG25 cellular localization was predicted using LncLocator, iLoc-LncRNA, and RNALocate, and the results showed that LncMSTRG25 tends to be located in the cytoplasm. In addition, after the mRNA level of LncMSTRG25 was knocked down using small interfering RNA (siRNA), LncMSTRG25 was labeled using specific probes, and RNA fluorescence in situ hybridization (FISH) results showed that LncMSTRG25 was localized in the cytoplasm (Fig. 3G). Taken together, the findings demonstrated the successful identification of hBMSCs and showed expression changes in LncMSTRG25, PAX8, and miR-939-5p during the differentiation of hBMSCs into osteoblasts.

### LncMSTRG25 knockdown inhibits osteogenic differentiation of hBMSCs

**Fig. 4.**
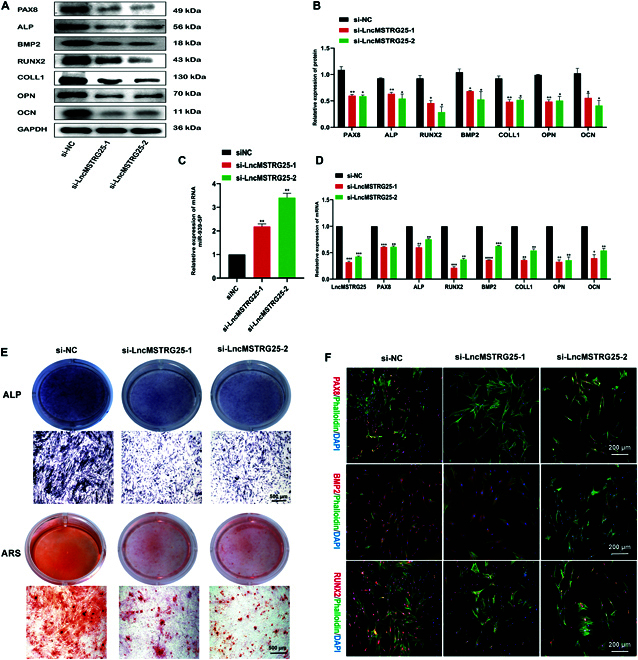
LncMSTRG25 knockdown inhibited osteogenic differentiation of hBMSCs. (A and B) hBMSCs were transfected with siLncMSTRG25-1, siLncMSTRG25-2, and the negative control and induced to differentiate into osteoblasts for 7 d. To evaluate protein expression levels, Western blotting was performed like PAX8, ALP, BMP2, RUNX2, COLL1, OPN, and OCN. (C and D) qPCR was used to detect the relative expression levels of miR-939-5p, LncMSTRG25-1, PAX8, ALP, BMP2, RUNX2, COLL1, OPN, and OCN. (E) hBMSCs were transfected with siLncMSTRG25-1, siLncMSTRG25-2, or a negative control and induced to differentiate into osteoblasts for 14 d. ARS and ALP staining methods were utilized to identify osteoblast differentiation. (F) Immunofluorescence with specific antibodies was used to detect the expression of PAX8, BMP2, and RUNX2 following 7 d of osteoblast differentiation. Data are the mean ± SD of 3 independent experiments. *P < 0.05, **P < 0.01, ***P < 0.001, and ****P < 0.0001.

To further verify the role of LncMSTRG25, we designed 3 specific siRNAs targeting LncMSTRG25, and through qPCR, we chose 2 siRNAs with knockdown efficiency. We transfected siLncMSTRG25-1, siLncMSTRG25-2, and NC (Negative Control) into hBMSCs using lip3000, and PCR assay showed that LncMSTRG25 expression showed a marked decrease relative to the NC control. At the same time, the expression levels of PAX8 and osteogenic markers, including ALP, BMP2, RUNX2, COLL1, OPN, and OCN, were detected, and the results showed that LncMSTRG25 knockdown effectively inhibited osteogenic-related genes at the mRNA and protein levels (Fig. 4A to D). Next, we continued the osteogenic differentiation of hBMSCs for 14 d, and in vitro osteogenic potential was assessed using ARS and ALP (alkaline phosphatase) staining. Interestingly, the ARS and ALP intensities were significantly lower in LncMSTRG25 knockdown hBMSCs than they were in the NC control, reflecting reduced osteogenic differentiation potential (Fig. 4E). We induced osteogenic differentiation of hBMSCs for 3 d and analyzed the expression of PAX8, BMP2, and RUNX2 using immunofluorescence. A significant decrease in PAX8, BMP2, and RUNX2 fluorescence intensities was observed in siLncMSTRG25-treated hBMSCs relative to the NC control group (Fig. 4F). These data suggest that LncMSTRG25 is involved in hBMSC differentiation during development and plays a positive adjustment role.

### Overexpression of LncMSTRG25 promotes osteogenic differentiation of hBMSCs

**Fig. 5.**
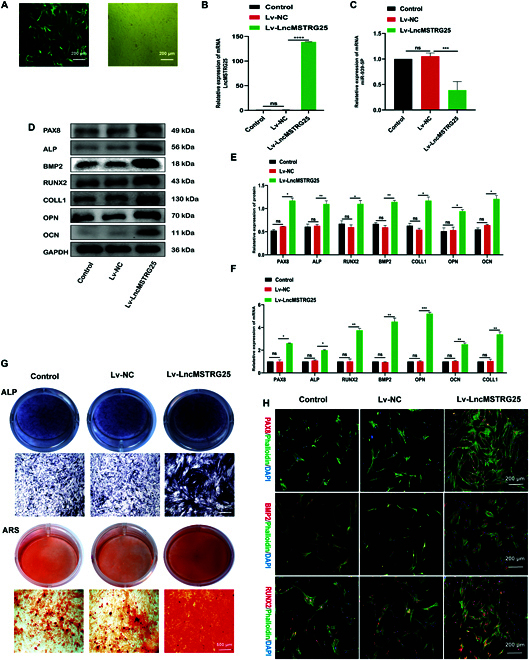
LncMSTRG25 overexpression promotes osteogenic differentiation of hBMSCs. (A) Fluorescent images showing the transfection efficiency of Lv-LncMSTRG25. (B,C, and F) PCR was conducted to evaluate the relative expression of LncMSTRG25-1, miR-939-5p, PAX8, ALP, BMP2, RUNX2, COLL1, OPN, and OCN. (D and E) Lv-LncMSTRG25 and Lv-NC were transfected into hBMSCs and induced to differentiate into osteoblasts for 7 d. Protein expression levels of PAX8, ALP, BMP2, RUNX2, COLL1, OPN, and OCN were assessed using Western blotting. (G) Lv-LncMSTRG25 and Lv-NC were transfected into hBMSCs and induced to differentiate into osteoblasts for 14 d. ARS and ALP staining were used to detect osteoblast differentiation. (H) Expression of PAX8, BMP2, and RUNX2 was detected by immunofluorescence staining with specific antibodies after 7 d of osteoblast differentiation. Results are presented as the mean ± SD of 3 independent trials, with significance levels of *P < 0.05, **P < 0.01, ***P < 0.001, and ****P < 0.0001.

Lv-LncMSTRG25 and Lv-NC were transfected into hBMSCs to study the osteogenic regulatory effect of LncMSTRG25 overexpression on hBMSCs. The MOI (multiplicity of infection) selection for hBMSCs is shown (Fig. 5A). The positive proportion of green fluorescent cells reached >90% in the MOI = 5 group. Accordingly, an MOI of 5 was chosen for the following experiments. According to the results of the Lv-LncMSTRG25 transfection group, LncMSTRG25 expression was approximately 130 times (Fig. 5B) and significantly inhibited compared with that of LncMSTRG25 miR-939-5p (Fig. 5C). Lv-LncMSTRG25 significantly increased the expression levels of mRNA and proteins for PAX8 and osteogenic markers (ALP, BMP2, RUNX2, COLL1, OPN, and OCN) in hBMSCs when compared with those of Lv-NC. (Fig. 5D to F). The results of ALP and ARS staining showed that Lv-LncMSTRG25 significantly promoted ALP activity and calcium deposition in hBMSCs undergoing osteogenic differentiation for 14 d (Fig. 5G). In addition, the results of cellular immunofluorescence and phalloidinium staining showed that the fluorescence intensity of PAX8, BMP2, and RUNX2 was significantly increased in Lv-LncMSTRG25-transfected hBMSCs compared with that in Lv-NC-transfected hBMSCs (Fig. 5H). These results imply that Lv-LncMSTRG25 substantially enhanced osteogenic differentiation in hBMSCs.

### Effect of miR-939-5p on osteogenic differentiation of hBMSCs

**Fig. 6.**
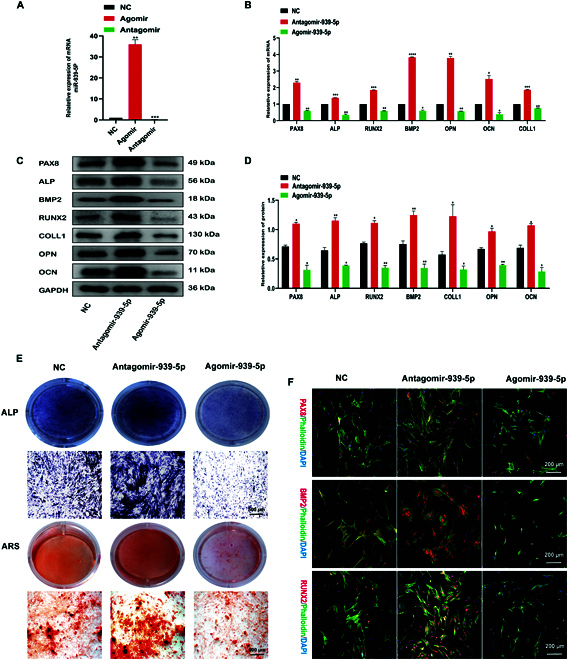
Osteogenic effects of miR-939-5p on hBMSCs. (A and B) PCR analysis was conducted to measure the relative expression of miR-939-5p, PAX8, ALP, BMP2, RUNX2, COLL1, OPN, and OCN. (C and D) hBMSCs were transfected with the miR-939-5p agomir, antagomir, or NC, and induced to differentiate into osteoblasts for 7 d. Western blotting was carried out to measure the protein expression of PAX8, ALP, BMP2, RUNX2, COLL1, OPN, and OCN. (E) hBMSCs were transfected with miR-939-5p agomir, antagomir, or NC and induced to differentiate into osteoblasts for 14 d. ARS and ALP staining was used to detect osteoblast differentiation. (F) hBMSCs were transfected with specific miRNA for 7 d, and immunofluorescence analysis was performed to detect the expression of PAX8, BMP2, and RUNX2. Results are presented as the mean ± SD of 3 independent trials, with significance levels of *P < 0.05, **P < 0.01, ***P < 0.001, and ****P < 0.0001.

To verify miR-939-5p in hBMSCs during osteogenetic differentiation, we compared miR-939-5p agomir or antagomir transfection of hBMSCs with that of the NC agomir and antagomir groups, which were approximately 10 and 30 times higher, respectively (Fig. 6A). The expression of mRNA and proteins was analyzed using PCR and Western blotting, and the results demonstrated that the antagomir significantly increased the expression of PAX8 and key osteogenic markers at both the mRNA and protein levels in hBMSCs, whereas the agomir had the opposite effect (Fig. 6B to D). At the same time, ALP and ARS staining showed that 14 d of osteogenesis-induced inhibition of miR-939-5p promoted osteogenesis, whereas miR-939-5p inhibited osteogenesis (Fig. 6E). Cellular immunofluorescence and phalloidin staining yielded similar results. Fluorescence intensities of PAX8, BMP2, and RUNX2 were significantly elevated in hBMSCs transfected with the miR-939-5p antagomir, in contrast to the NC group; conversely, hBMSCs transfected with the miR-939-5p agomir exhibited the opposite pattern (Fig. 6F). Taken together, our data support that miR-939-5p has a notable impact on the osteogenic differentiation of hBMSCs.

### miR-939-5p is the downstream target of Lv-LncMSTRG25-mediated osteogenic differentiation of hBMSCs

**Fig. 7.**
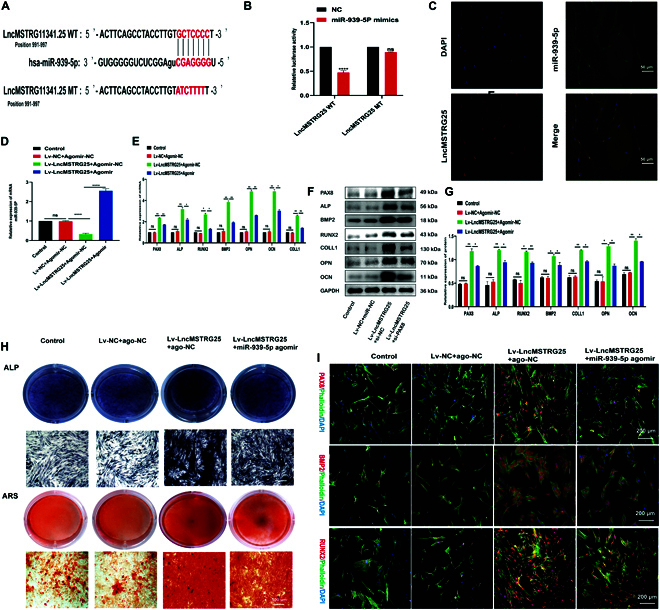
LncMSTRG25 has miR-939-5p binding targets, and the interaction affects osteogenic differentiation. (A) Using miReap, miRanda, and TargetScan tools, we predicted potential binding sites between LncMSTRG25 and miR-939-5p, and validated these predictions through a dual-luciferase reporter assay. (B) Cotransfection of the miR-939-5p plasmid and LncMSTRG25-WT plasmid significantly decreased luciferase activity, as shown by dual-luciferase reporter gene analysis. (C) FISH data showed that LncMSTRG25 and miR-939-5p were both present in the cytoplasm of hBMSCs. (D and E) Lv-LncMSTRG25, Lv-NC, and miR-939-5p mimics or miRNA negative controls were cotransfected into hBMSCs. PCR analysis was conducted to measure the relative expression of PAX8, ALP, BMP2, RUNX2, COLL1, OPN, and OCN following 7 d of osteogenic differentiation induction. (F and G) The protein expression levels of PAX8, ALP, BMP2, RUNX2, COLL1, OPN, and OCN were determined by Western blotting. (H) Osteogenic differentiation was carried out for 14 d, and ARS and ALP staining was performed to determine the differentiation. (I) Following 7 d of osteogenic differentiation, the expression of PAX8, BMP2, and RUNX2 was analyzed by immunofluorescence staining with specific antibodies. Results are presented as the mean ± SD of 3 independent trials, with significance levels of *P < 0.05, **P < 0.01, ***P < 0.001, and ****P < 0.0001.

Bioinformatics predictions suggest that LncMSTRG25 may be a target of miR-939-5p. Figure 7A shows the predicted bioinformatics binding sites between the 2 mRNAs. The dual-luciferase reporter assay further demonstrated that cotransfection with miR-939-5p mimics significantly decreased the relative luciferase activity of LncMSTRG25-WT when compared with the NC group. However, in the presence of miR-939-5p mimics, the luciferase activity of LncMSTRG25-MUT carrying the miR-939-5p binding site mutation remained largely unchanged (Fig. 7B). To examine the role of LncMSTRG25 in the osteogenic differentiation process controlled by miR-939-5p, we first used the coding LncMSTRG25 lentivirus transfection of hBMSCs LncMSTRG25 to stabilize its expression. Cotransfection with Lv-LncMSTRG25 and miR-939-5p mimics partially restored miR-939-5p levels in hBMSCs (Fig. 7C). Next, we assessed the effects of Lv-LncMSTRG25 and miR-939-5p agomir on the osteogenic differentiation ability of hBMSCs. PCR and Western blotting validation showed that in the process of osteogenic differentiation, compared with hBMSCs transfected with Lv-LncMSTRG25, hBMSCs cotransfected with Lv-LncMSTRG25 and miR-939-5p mimics saw reduced expression of the target gene PAX8 and osteogenesis-related genes, including ALP, BMP2, RUNX2, COLL1, OPN, and OCN (Fig. 7D to F). Using FISH, we further verified that LncMSTRG25 and miR-939-5p in hBMSCs were located in the cytoplasm (Fig. 7G). At the same time, compared with the transfection of Lv-LncMSTRG25 hBMSCs, hBMSCs cotransfected with Lv-LncMSTRG25 and miR-939-5p mimics showed reduced ALP and ARS staining intensities (Fig. 7H). Immunofluorescence results for PAX8, BMP2, and RUNX2 in hBMSCs showed a similar trend (Fig. 7I). Our results show that miR-939-5p partially blocks the osteopromotive effect of LncMSTRG25. These findings imply that LncMSTRG25 regulates osteogenesis by interacting with miR-939-5p.

### Effect of PAX8 on osteogenic differentiation of hBMSCs

**Fig. 8.**
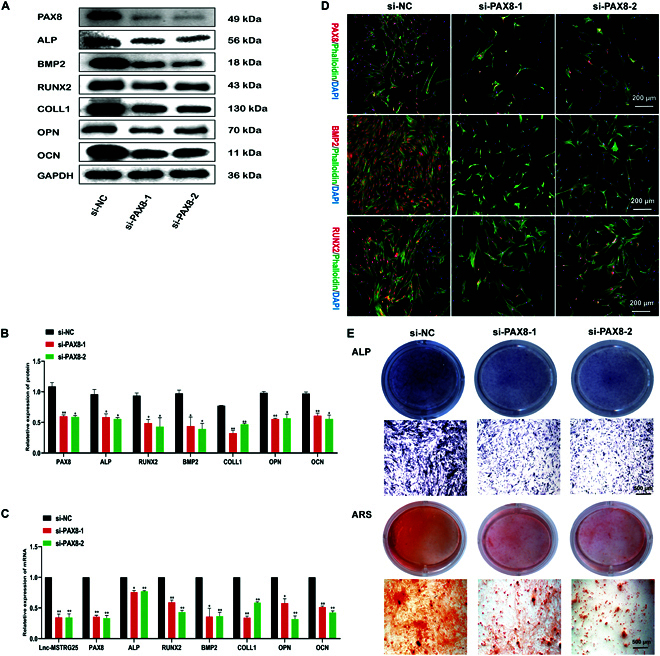
PAX8 knockdown inhibits bone formation of hBMSC. (A and B) siPAX8-1, siPAX8-2, and NC were transfected into hBMSCs and induced to differentiate into osteoblasts for 7 d. The protein expression levels of PAX8, ALP, BMP2, RUNX2, COLL1, OPN, and OCN were determined by Western blotting. (C) PCR analysis was conducted to measure the relative expression of LncMSTRG25-1, miR-939-5p, PAX8, ALP, BMP2, RUNX2, COLL1, OPN, and OCN. (D) The expression of PAX8, BMP2, and RUNX2 was assessed by immunofluorescence after 7 d of osteoblast differentiation. (E) siPAX8-1, siPAX8-2, and NC were transfected into hBMSCs, and the cells underwent osteoblast differentiation for 14 d. ARS and ALP staining was employed to evaluate the differentiation. Results are presented as the mean ± SD of 3 independent trials, with significance levels of *P < 0.05, **P < 0.01, ***P < 0.001, and ****P < 0.0001.

To further investigate the role of PAX8 in osteogenic differentiation of hBMSCs, we designed 3 siRNAs specifically targeting PAX8. We used lip3000 to transfect siPAX8 into hBMSCs, and verified the results by PCR. Two siRNAs with the highest knockdown efficiencies were selected, and PAX8 expression was significantly reduced. We then examined osteogenic markers, including ALP, BMP2, RUNX2, COLL1, OPN, and OCN, at the mRNA and protein levels, and the results showed that PAX8 knockdown effectively inhibited osteogenesis-related gene expression (Fig. 8A to C). We induced osteogenic differentiation of hBMSCs for 3 d and analyzed the expression of PAX8, BMP2, and RUNX2 using immunofluorescence. Compared with the NC control group, the siPAX8, PAX8, BMP2, and RUNX2 groups decreased (Fig. 8D). Next, we proceeded to osteogenic induction of hBMSCs (as described above) for 14 d and evaluated their osteogenic differentiation potential in vitro using ARS and ALP staining. Similarly, compared with the NC group, PAX8 knockdown hBMSCs showed lower ARS and ALP intensities, indicating that the ability of hBMSCs to differentiate osteogenically was impaired after PAX8 knockdown (Fig. 8E). These data support the notion that PAX8 is an important regulator of osteogenic differentiation in hBMSCs.

### LncMSTRG25 up-regulated PAX8 by direct binding to miR-939-5p, and knockdown of PAX8 attenuated the effect of LncMSTRG25 on promoting osteogenic differentiation of hBMSCs

**Fig. 9.**
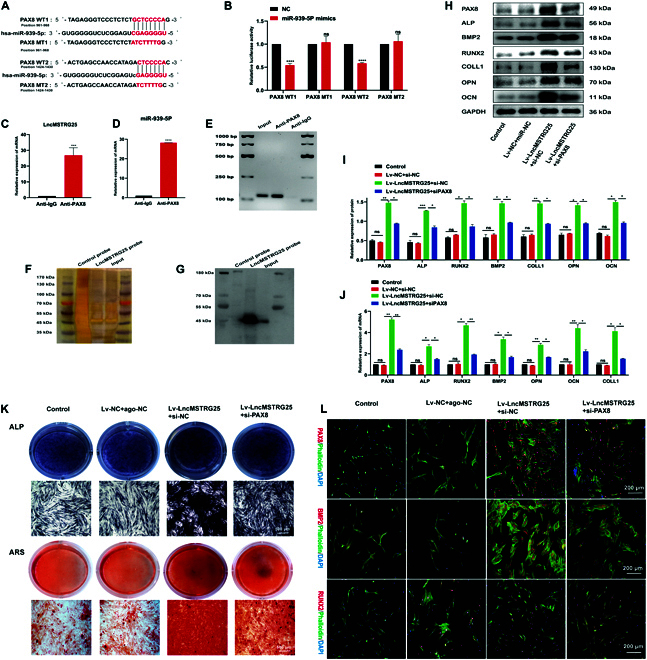
LncMSTRG25–PAX8–miR-939-5p regulatory axis regulates hBMSC osteogenesis. (A) Potential target-binding sites of PAX8 and miR-939-5p were predicted using TargetScan and StarBase, and then validated through a dual-luciferase reporter assay. (B) The results from the dual-luciferase reporter gene assay indicated that cotransfection with the miR-939-5p plasmid and PAX8-WT1 and PAX8-WT2 plasmids caused a significant decrease in luciferase activity. (C to E) RNA RIP experiments were performed on hBMSCs using PAX8 antibody to evaluate the enrichment effect of anti-PAX8 on LncMSTRG25 and miR-939-5p. qPCR was used to detect RNA levels in the immunoprecipitates. The miR-939-5p level in the anti-PAX8 group was higher compared with that in the anti-IgG group, and the level of LncMSTRG25 was significantly increased in the anti-PAX8 group compared with that in the anti-IgG group. The LncMSTRG25 PCR product was subjected to agarose electrophoresis, and in the anti-PAX8 group, the expression level was markedly increased. (F and G) Silver staining and Western blotting results of RNA pull-down samples showed that the biotin-labeled LncMSTRG25-specific probe group significantly enriched PAX8. (H and I) Using Lv-LncMSTRG25, Lv-NC, siPAX8, or an siRNA negative control, osteogenic differentiation was induced for 7 d. Protein expression levels of PAX8, ALP, BMP2, RUNX2, COLL1, OPN, and OCN were analyzed by Western blotting. (J) To detect the relative expression levels of PAX8, ALP, BMP2, RUNX2, COLL1, OPN, and OCN, PCR was performed. Osteogenic differentiation continued for 14 d. (K) ARS and ALP staining were used to monitor osteogenic differentiation. (L) Specific antibody immunofluorescence staining was used to detect the expression of PAX8, BMP2, and RUNX2 after 7 d of osteogenic differentiation. Results are presented as the mean ± SD of 3 independent trials, with significance levels of *P < 0.05, **P < 0.01, ***P < 0.001, and ****P < 0.0001.

To predict and verify the potential of miR-939-5p downstream targets, we analyzed the RNAInter database and identified miR-939-5p and PAX8 between the 2 binding sites (Fig. 9A). PAX8-WT1 and PAX8-WT2 were labeled as the binding sites and verified using dual-luciferase reporter plasmids. Relative to the NC group, transfection with miR-939-5p mimics and PAX8-WT significantly decreased luminous intensity. Next, after cotransfection with miR-939-5p mimics, we constructed PAX8-MUT carrying miR-939-5p binding site mutations, labeled as PAX8-MUT1 and PAX8-MUT2. Luciferase activity remained unchanged following cotransfection with miR-939-5p mimics (Fig. 9B). The data imply that miR-939-5p has the potential to interact with both of the predicted binding sites, PAX8 sites 1 and 2. Next, we performed RNA immunoprecipitation (RIP) experiments to explore whether LncMSTRG25 is associated with PAX8. PCR by immunoprecipitation revealed a significant enrichment (25-fold up-regulation) of LncMSTRG25 in the PAX8 group (anti-PAX8) compared with that in the control immunoglobulin G (anti-IgG) group (Fig. 9C). At the same time, in immunoprecipitates containing PAX8, the levels of miR-939-5p were much higher (by 29 times) than those in IgG immune precipitation (Fig. 9D). PCR products further analyzed by agarose electrophoresis showed the anti-PAX8 group enrichment of LncMSTRG25 (Fig. 9E), suggesting that LncMSTRG25 of miR-939-5p has a sponge effect and there is interaction among all 3. Next, we used RNAplex [22,23] to predict whether PAX8 is a potential target of LncMSTRG25. A biotin-labeled LncMSTRG25-specific probe was designed for further validation, and an RNA pull-down assay was performed on hBMSCs. Enrichment of silver staining and Western blotting showed PAX8 protein bands (Fig. 9F to G). To further clarify LncMSTRG25 and PAX8 on osteogenic differentiation, we used PCR and Western blotting to verify PAX8 and osteogenic markers. Lv-LncMSTRG25 and siPAX8 were cotransfected; compared with hBMSCs transfected with Lv-LncMSTRG25, hBMSCs cotransfected with Lv-LncMSTRG25 and siPAX8 showed that the up-regulation of PAX8, ALP, BMP2, RUNX2, COLL1, OPN, and OCN caused by LncMSTRG25 overexpression was inhibited to varying degrees (Fig. 9H to J). The intensity of ALP and ARS staining was significantly weaker than that in the Lv-LncMSTRG25 group (Fig. 9K), indicating that the promotion of osteogenesis by LncMSTRG25 could be partially blocked by siPAX8. Finally, PAX8, BMP2, and RUNX2 immunofluorescence results showed similar trends (Fig. 9L). Collectively, our results indicate that the osteogenic differentiation-promoting activity of LncMSTRG25 is partially regulated by PAX8 knockdown.

## Discussion

Recent studies increasingly indicate that the immune microenvironment is essential to fracture healing [24]. Our previous study found that patients with fractures and splenectomy had significantly reduced levels of proinflammatory factors in the initial stages of bone fracture healing, and that proinflammatory factors are essential for the early stages of fracture healing [5,6]. Studies have revealed that the immune inflammatory microenvironment modulates the osteogenic differentiation of MSCs. IL-1β and TNF-α play roles in the osteogenic differentiation of MSCs through different signaling pathways [8,20,25]. Based on our previous research results, we used IL-1β to treat hBMSCs in vitro in order to identify new lncRNA. When the data were sufficient, multi-omics analysis was performed to screen and verify all lncRNAs that may participate in the physiological process of hBMSC differentiation into osteogenic cells, and to identify new lncRNAs with high expression levels. We screened dozens of new lncRNAs related to osteogenic differentiation using RNA sequencing analysis and observed significant up-regulation of LncMSTRG25 during hBMSC differentiation into osteoblasts, while silencing LncMSTRG25 inhibited bone formation, confirming the significant function of the LncMSTRG25–miR-939-5p–PAX8 axis. We also observed significant changes in the expression of osteogenesis-related genes, including SOD2 and ADAM12 (Fig. 2). MSCs are attractive for the treatment of bone diseases and have presented promising benefits in particular clinical practices, such as fracture healing and bone tissue engineering [26,27]. However, further investigation is needed to better understand the regulatory mechanisms of the immune system on osteoblast differentiation of hBMSCs after splenectomy and provide credible theoretical insight for future clinical practices.

Increasing evidence suggests that lncRNAs play key roles in epigenetic modifications, transcriptional expression, and posttranscriptional regulation [28,29]. The most well-known theory is that lncRNAs function as miRNA “sponges”. Moreover, they have attracted increasing attention because of their close relationship with the osteogenesis of MSCs and their potentially important role in various bone diseases [30,31]. However, the mechanisms by which lncRNAs participate in osteogenesis remain unclear. Given the stability of the abundance ratio of lncRNAs and miRNAs and the number of miRNA binding sites in lncRNAs, this contributes to the interaction between lncRNA and miRNA “sponges” [32,33]. Considering the abundance of lncRNA and number of binding sites, we speculated that LncMSTRG25 and miR-939-5p have an interactive “sponge” role in hBMSCs. Stem cell studies have validated a similar model. For instance, a study reported that LNC_000052 “sponge” miR-96-5p regulates the behavior of rat BMSCs [34], while LncMGR contains miR-2131-5p binding targets that regulate muscle cell development [35]. In our study, bioinformatic analysis using miReap, miRanda, and TargetScan showed that miR-939-5p, which is down-regulated during hBMSC osteogenesis, has binding targets for LncMSTRG25 and PAX8. The function of miR-939-5p in hBMSCs has not been documented, and our findings indicate that miR-939-5p can enhance osteogenesis. The binding sites of LncMSTRG25 and miR-939-5p, along with PAX8 and miR-939-5p, were validated using dual-luciferase reporter assays. The qPCR results demonstrated that silencing miR-939-5p enhances the expression of PAX8, whereas the overexpression of LncMSTRG25 up-regulates the expression of PAX8. We conclude that PAX8 is a target of miR-939-5p and that LncMSTRG25 is a ceRNA sponge for miR-939-5p. Therefore, LncMSTRG25 regulates the osteogenic differentiation of hBMSCs by acting on miR-939-5p through a sponge function, whereas miR-939-5p prevents osteogenic differentiation of hBMSCs by down-regulating PAX8 translation. The LncMSTRG25–miR-939-5p–PAX8 axis, together with its targets, may have an extensive regulatory gene expression network; however, further investigation is essential to elucidate the mechanisms involved.

PAX8 is part of the paired box transcription factor family, which is crucial for embryonic development and cell differentiation. During organ and tissue development, PAX8 is crucial for cell differentiation, proliferation, and migration [36,37]. It is essential in sustaining the functional differentiation of thyroid cells, can delay cardiomyocyte apoptosis, and promotes cardiomyocyte development [38–40]. Mouse cardiomyocytes lacking the transcription factor PAX8 show developmental and apoptosis defects. Moreover, research has demonstrated that PAX8 is strongly associated with bone formation and is up-regulated in osteoporotic mice [41,42]. In this study, LncMSTRG25 significantly promoted the expression of PAX8 and osteogenesis, whereas knockdown of PAX8 inhibited osteogenesis. Our study showed that sustained up-regulation of PAX8 is essential in facilitating the differentiation of hBMSCs into osteoblasts.

MiRNA is a family of small noncoding RNA molecules, 21 to 25 nucleotides in length, that play a significant regulatory role in the osteoblast differentiation of stem cells, such as miR-335-5p, miR-200, and miR-26b. Their regulatory function is primarily carried out by binding to the 3′UTR of the target gene. They silence gene expression by inhibiting translation or cleaving mRNA, playing a crucial role in posttranscriptional regulation of gene expression [43–45]. Growing research indicates that miRNAs modulate bone development through the targeting of specific transcription factors and cellular signaling pathways, such as the BMP2, WNT, and SMAD pathways [46,47]. We found that miR-939-5p expression was markedly reduced during the differentiation of hBMSCs into osteoblasts. Additionally, we validated the competitive interaction between LncMSTRG25 and miR-939-5p. MiR-939-5p overexpression blocked the osteogenesis of hBMSCs by increasing the expression of LncMSTRG25. Dual-luciferase assay further verified the binding of the 2 proteins. Studies have revealed that lncRNA can function as strong sponges for miRNA that affect gene transcription and translation; in addition, they bind to specific miRNA and relieve their inhibitory activity on target mRNA.

In our research, we identified a new molecular network, composed of LncMSTRG25, miR-939-5p, and PAX8, which regulates hBMSC osteogenesis. We verified the effects of overexpression and knockdown of LncMSTRG25, miR-939-5, and PAX8 on osteogenesis observed in previous in vitro experiments. However, using the NONCODE database, we predicted that LncMSTRG25 has little homology with other animals, which limits in vivo animal experiments. Future work is needed to address these and other issues, including further in vivo verification of the effects of LncMSTRG25, miR-939-5p, and PAX8 on osteogenesis and the involvement of other lncRNA–miRNA–mRNA axes in osteogenesis after splenectomy. Simultaneously, the therapeutic effects of hBMSCs should be observed with the combined use of x-ray imaging, micro-computed tomography, or intravenous injection of lncRNA-modified hBMSCs. This method has been widely used in laboratories and clinical trials and could be used to explore the in vivo effects of LncMSTRG25 more comprehensively. Our findings provide new insight into the fine regulatory network of lncRNA–miRNA–mRNA in the bone immune microenvironment after splenectomy. LncRNA-based therapy requires a deep understanding of its multiple functions in gene regulation. At the same time, owing to the complex microenvironment of the human body, its efficacy is affected by multiple factors such as body homeostasis, mechanical stress, and the immune microenvironment [48], which requires further study in the future.

## Materials and Methods

### Extraction and identification of hBMSC

We isolated hBMSCs from the bone marrow of patients with limb-long bone fractures; all procedures were performed according to the ethical review board approval of the Union Hospital Affiliated to Huazhong University of Science and Technology (UHCT-IEC-SOP-016-03-01). hBMSCs from 3 donors (average age, 28 years) were cultured in a Dulbecco’s modified Eagle’s medium/F12 (Gibco, USA), 15% fetal bovine serum (ExCell Bio, China), and 1% penicillin–streptomycin (Beyotime, China) growth medium. hBMSCs at a density of 1 × 10^5^ cells/cm^2^ were seeded in medium-sized cell culture dishes, and the culture conditions were 37 °C and 5% CO_2_. The medium was refreshed every 3 d. Separation was based on a previously reported protocol for hBMSCs [49]. The differentiation of hBMSCs was induced using IL-1β (5 ng/ml) (MedChemExpress, USA). Flow cytometry was used to sort and identify specific markers of hBMSCs, CD19, CD34, CD45, CD44, CD90, and CD105 (all from BD, USA).

### Transfection of hBMSCs

GenePharma (Suzhou, China) was responsible for the design and synthesis of the miR-939-5p antagomir, miR-939-5p mimics, siRNA, siLncMSTRG25,siPAX8, miR-939-5p agomir, and a negative control. The sequences are shown in Table S1. We used Lipofectamine 3000 plasmid transfection reagent (Invitrogen, Carlsbad, CA, USA) and the corresponding negative controls. In slow-virus infection, LncMSTRG25 induced a slow virus and was provided by Genechem (Shanghai, China). A density of 5 × 10^4^ cells/ml was used to seed hBMSCs in 6-well plates, and once the cells reached 70% confluence, they were infected with lentivirus at an MOI of 5. Lv-NC is an empty lentiviral system lacking insertion sequences.

### RNA sequencing analysis

Following total RNA extraction, ribosomal RNA was removed to enhance the retention of all coding RNA and lncRNA. RNA was randomly fragmented into short lengths, and the resulting fragments served as a template for first-strand cDNA synthesis using random hexamers. The second-strand cDNA was synthesized by adding buffer, deoxynucleotide triphosphates (with dUTP replacing dTTP), ribonuclease (RNase) H, and DNA polymerase I. The purified product was obtained using a QiaQuick PCR kit, eluted with EB buffer, and then processed with end repair, base A addition, and sequencing adapter ligation. Uracil-N-glycosylase was employed to degrade the second strand. Fragment size selection for PCR amplification was carried out through agarose gel electrophoresis. Sequencing was performed on Illumina HiSeq 4000, and GeneDenovo conducted the data analysis (Biotechnology Co. Ltd., Guangzhou, China).

### ALP staining

Third-generation hBMSCs were grown in an osteogenic differentiation medium for 7 d (Cyagen, USA). At 3-d intervals, the medium was refreshed, and after induction, the cells were fixed with 4% paraformaldehyde. To evaluate the differentiation ability, ALP staining kits from Beyotime (China) were employed. According to the manual configuration of the ALP staining fluid, the cells were incubated with light for 30 to 60 min. The cells were observed and photographed under a light microscope.

### ARS staining

For 14 d, the third-generation hBMSCs were cultured in osteogenic differentiation induction medium provided by Cyagen (USA); medium changes were performed every 3 d. After induction, the hBMSCs were fixed with 4% paraformaldehyde. ARS dyeing liquid (Solarbio, China) mineralization nodule staining was used to evaluate osteogenic differentiation ability. The samples were subjected to manual dyeing for 30 min, visualized under an optical microscope, and documented with photographs (Nikon, Tokyo, Japan).

### Immunofluorescence and cytoskeleton staining

Third-generation hBMSCs were planted on 12-well culture plates, mounted on a chip, and then allowed to develop in the osteogenetic differentiation medium for 3 d. The expression levels of PAX8, BMP2, and RUNX2 were assessed through immunofluorescence staining. Briefly, cells were fixed at the end of induction with 4% formaldehyde, followed by membrane disruption with 0.5% Triton. Cells were subsequently washed 3 times with PBS. Blocking of nonspecific cell surface binding was performed using goat serum. PAX8 was diluted 1:50 with an antibody diluent (Proteintech, China, 10336-1-AP). RUNX2 (Affinity China, AF5186) and BMP2 (Affinity China, AF5163) were diluted by 1:100, added to the 12-well culture plates, and then incubated at 4 °C overnight. Three washes with TBST (tris-buffered saline–Tween 20) were performed on the cells, and they were then maintained at room temperature with Alexa Fluor 594 mark of goat anti-rabbit secondary antibody (Proteintech, China, RGAR004) before being incubated at 37 °C in the dark for 2 h. Three washes with phosphate-buffered saline (PBS) were performed on the cells at room temperature. To each well, 150 μl of prepared fluorescein isothiocyanate-labeled phalloidin working solution (Solarbio, China) was added. Cells on coverslips were covered, and incubation was carried out in the dark at room temperature for 30 min. Finally, nuclei were stained with 50 μl of blue skies 4′,6-diamidino-2-phenylindole (DAPI) (Beyotime, China) and incubated in the dark at room temperature for 6 min, followed by 3 PBS washes. The slides were removed and placed on slides, and half a drop of an anti-fluorescence quenching agent was added. The slides were sealed with a coverslip and photographed using a fluorescence camera in a dark room (Nikon, Japan).

### RNA extraction and quantitation

RNA from hBMSCs was isolated using Trizol reagent (Takara, China), and the One-Step qPCR kit was used for mRNA cDNA synthesis (11142ES60, YeaSen, China) and used cDNA Synthesis Kit (11148ES50, YeaSen, China) for microRNA; total RNA was reverse-transcribed into cDNA. For qPCR, the PCR kit (11201ES08, YeaSen, China) and a fluorescence PCR system were utilized (Bio-Read, USA, CFX Connect). To normalize mRNA and lncRNA levels, glyceraldehyde-3-phosphate dehydrogenase (GAPDH) was used as the internal reference, while U6 small nuclear RNA (snRNA) was used for miRNA quantification. Finally, we calculated the relative expression of the target RNA by the 2^−ΔΔCt^ method. The design and synthesis of all primers were conducted by Sangon Biological (Shanghai, China). The sequences of the primers are shown in Table S2.

### Western blotting analysis

After 7 d of osteogenic differentiation of hBMSCs, cell lysates were prepared using RIPA (radioimmunoprecipitation assay) and protease inhibitors (Beyotime, China) for protein extraction. Protein concentration was quantified using a BCA assay kit from Beyotime (China). This was followed by sodium dodecyl sulfate–polyacrylamide gel electrophoresis on polyvinylidene difluoride membranes (Millipore, USA). Samples were blocked using 5% milk powder, followed by primary antibody incubation The primary antibodies used were against PAX8 (Proteintech China,10336-1-AP), OPN (Proteintech China, 22952-1-AP), COLL1 (Proteintech China, 28459-1-AP), OCN (Abclonal China, A20800), Runx2 (Affinity China, AF5186), BMP2 (Affinity China, AF5163), ALP (Affinity China, DF6225), and GAPDH (Proteintech China, 60004-1-Ig). Subsequently, samples were incubated at 4 °C overnight with the secondary antibody (Proteintech China, RGAR001). A chemiluminescence instrument was employed for exposure, and the ImageJ software was used to analyze the images (Bio-Rad, USA,1708370).

### Dual-luciferase reporter assay

Gnecreate (Wuhan, China) was used to design and synthesize recombinant dual-luciferase reporter vectors pmirGLO-LncMSTRG25-WT, pmirGLO-LncMSTRG25-MT, pmirGLO-PAX8-WT1, pmirGLO-PAX8-MT1, pmirGLO PAX8-WT2, pmirGLO PAX8-MT2, and miR-939-5p mimic miR-939-5p NC. hBMSCs were seeded in 96-well plates and cultured to 75% confluence. Lipofectamine 3000 (Invitrogen) was used as the luciferase reporter carrier, miR-939-5p analog, or its negative control (miR-NC). After 48 h, cells were harvested and the firefly and sea kidney luciferase activities were quantified using a dual-luciferase reporter system (Promega, WI, USA). The sequences are shown in Table S3.

### FISH experiments

FISH kits provided by Gemma (Suzhou, China) were used to detect the expression of LncMSTRG25 and miR-939-5p in the cytoplasm of hBMSCs. In brief, cells were washed at the end of the intervention in PBS and fixed in 4% formaldehyde, and fragmentation was carried out with 0.5% Triton X for 5 min at 4 °C. Subsequently, cells were incubated overnight in the dark at 37 °C for 30 min with 200 μl of degeneration-specific probe mixture added to each hole. Fluorescent tags LncMSTRG25 and miR-939-5p-specific probe were designed and synthesized using GenePharma (Suzhou, China). The Cy3-labeled LncMSTRG25 probe sequence was 5′-CTGAACTATGGAGAGGTGCACAAACCCATGTGTAGACTGATAGCTGTGCACAGTACCATT-3′. The FAM-labeled miR-939-5p probe sequence was 5′-CACCCCCAGAGCCTCAGCTCCCCA-3′. Fluorescent signals were detected using confocal microscopy after staining the nuclei with DAPI (Carl Zeiss, Germany).

### RNA immunoprecipitation

Following the manufacturer’s instructions, RIP experiments were conducted using the Magna RIP kit (Millipore, USA). Cells (2 × 10^7^) were collected, to which we added cell lysis buffer, protease inhibitors, and RNase inhibitor cell lysis solution; the samples were then centrifuged at 4 °C and 10,000*g*. The supernatant was removed, and 50 μl of cell lysate was used as the input for qPCR. We took 500 μl of cell lysis solution and added anti-PAX8 (Cell Signaling Technology, USA, D2S2I) magnetic beads or IgG (Proteintech, China, 30000-0-AP) immune coprecipitation magnetic beads. The samples were marked as IP (immunoprecipitation) and IgG groups, respectively. Then, the samples were subjected to a 10 rpm rotating reaction at 4 °C overnight. After thorough washing, the immunoprecipitated RNA was extracted. qPCR was used to detect immune RNA expression during coprecipitation. The PCR products were visualized using ethyl bromide dye on 2% agarose gel, and 6 × DNA sample buffer (Beyotime, China) was added.

### RNA pull-down

A Pierce Magnetic RNA-Protein Pull-Down Kit was utilized for performing RNA pull-down (Thermo Fisher Scientific). We used a biotin-labeled LncMSTRG25 probe sequence (5′-CAAACCCATGTGTAGACTGA-3′-biotin) and contrast probe (5′-TGCTTTGCACGGTAACGCCTGTTTT-3′-biotin). The LncMSTRG25 specificity of the biotin probe was designed and synthesized using GenePharma (Suzhou, China). To obtain cell lysates, the cell lysis buffer was combined with protease and RNase inhibitors and incubated on ice for 20 min. The sample was centrifuged at 12,000*g* and 4 °C to obtain the supernatant. After the pre-processing of streptavidin magnetic beads, we combined the tubes of magnetic beads with 50 pmol purpose probe or 50 pmol contrast probe, and then subjected them to a 10 rpm rotating reaction for 30 min at 4 °C. The fluid cell lysis supernatant was discarded, and then the sample was subjected to rotating incubation at 4 °C for 1 h. Samples were then washed to collect RNA magnetic bead compounds, and the final protein product was eluted. The obtained products were analyzed for PAX8 expression by Western blotting and silver staining.

### Statistical analysis

Quantitative variables are expressed as the mean ± standard deviation, and comparisons between the 2 groups were performed using a 2-way *t* test. For comparisons among multiple groups, one-way analysis of variance (ANOVA) was used, with the Bonferroni test for post hoc pairwise comparisons.

## Data Availability

The data that support the findings of this study are available from the corresponding author upon reasonable request.

## Supplementary Materials

Supplementary 1 Figure 2D Tables S1 to S3
