# Supplementary figures and images for: Novel lncRNA LncMSTRG.11341.25 Promotes Osteogenic Differentiation of Human Bone Marrow Stem Cells via the miR-939-5p/PAX8 Axis
Source: Research (Wash D C). 2025 Feb 6;8:0601. doi: 10.34133/research.0601 (PMC11798881; doi:10.34133/research.0601)

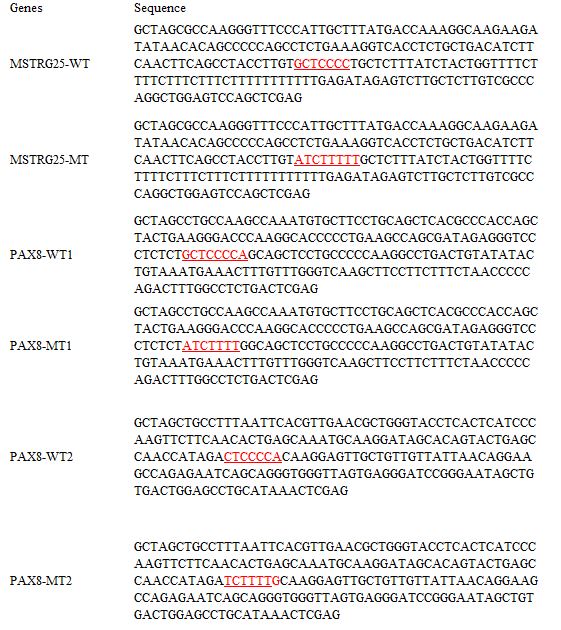

Supplement: Supplementary 1 — Figure 2D Tables S1 to S3 [file research.0601.f1.zip › Table 3.JPG]

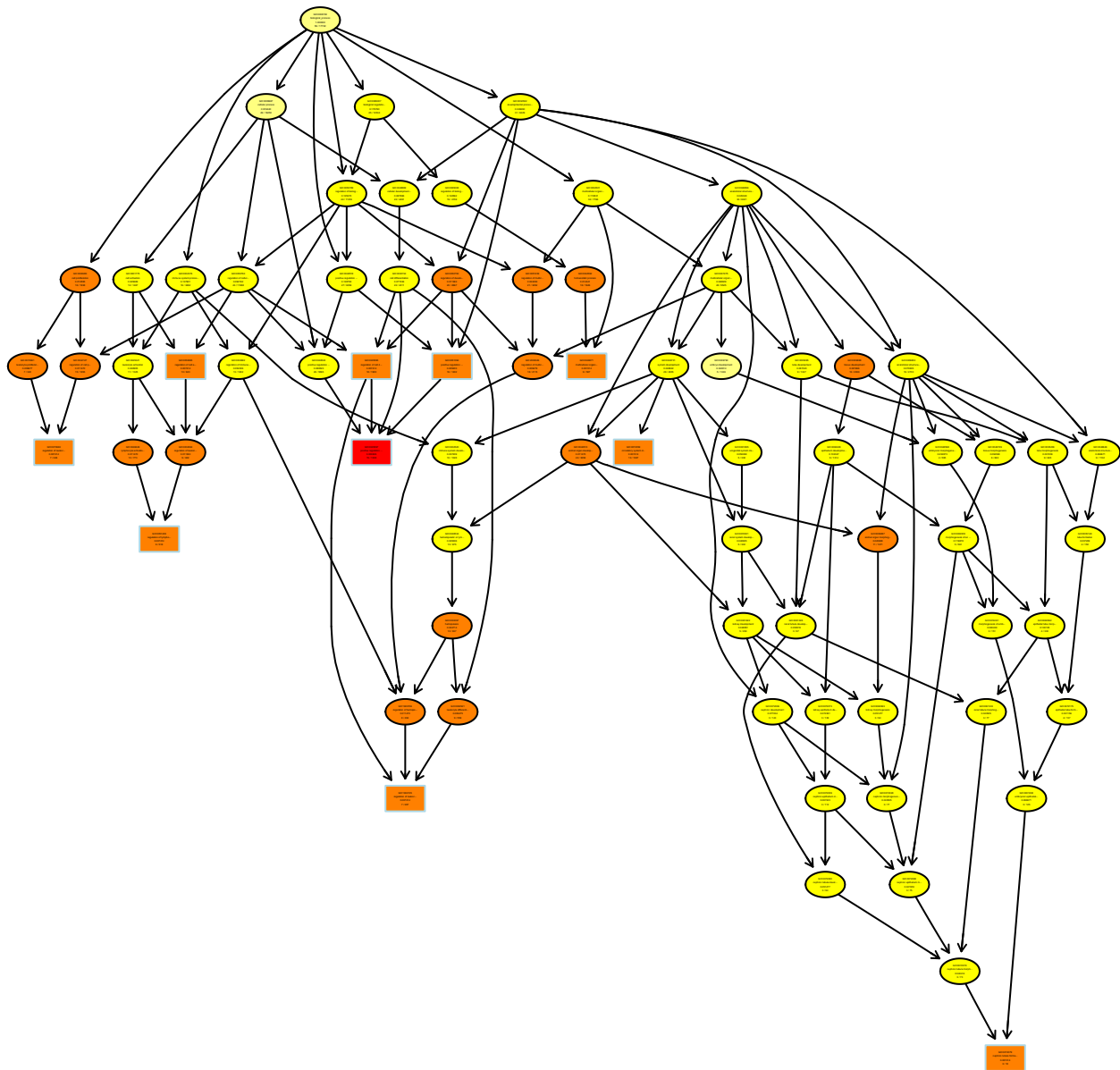

Supplement: Supplementary 1 — Figure 2D Tables S1 to S3 [file research.0601.f1.zip › Figure2D-1.pdf]

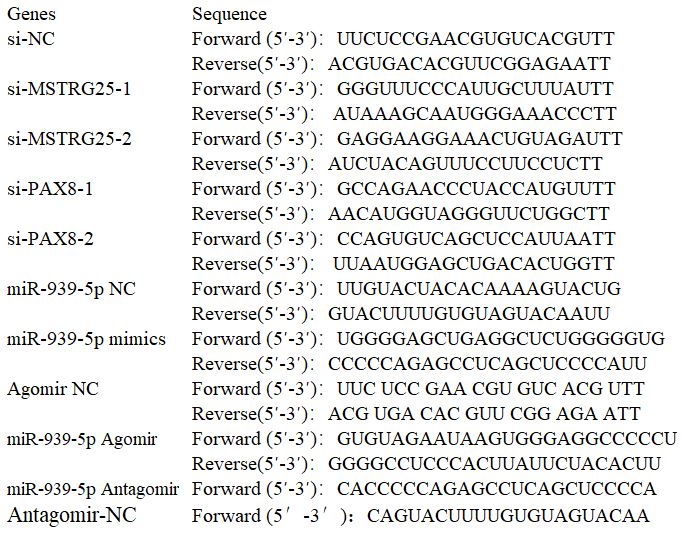

Supplement: Supplementary 1 — Figure 2D Tables S1 to S3 [file research.0601.f1.zip › Table 1.JPG]

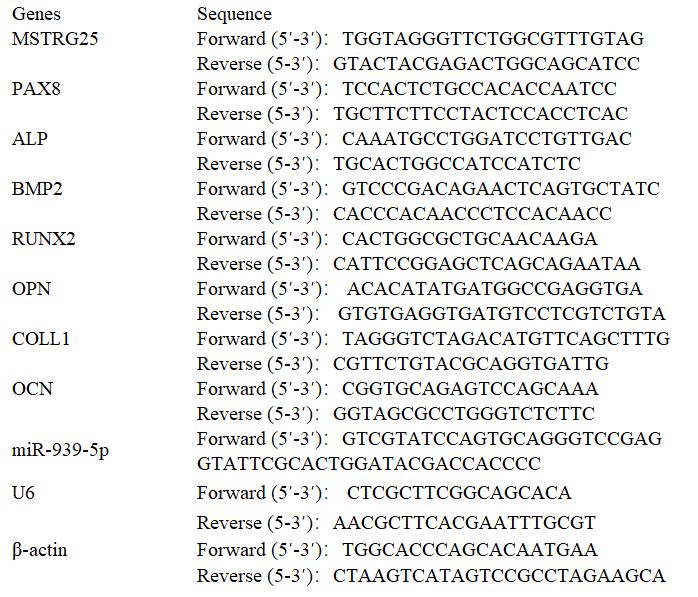

Supplement: Supplementary 1 — Figure 2D Tables S1 to S3 [file research.0601.f1.zip › Table 2.JPG]
